# Fenugreek Compound (N55) Lowers Plasma Glucose through the Enhancement of Response of Physiological Glucagon-like peptide-1

**DOI:** 10.1038/s41598-017-12290-x

**Published:** 2017-09-25

**Authors:** I-Wen Chou, Yu-Hong Cheng, Yet-Ran Chen, Patrick Ching-Ho Hsieh, Klim King

**Affiliations:** 1 Graduate Institute of Life Sciences, National Defense Medical Center, Taipei, 114 Taiwan; 2 Institute of Biomedical Sciences, Academia Sinica, Taipei, 115 Taiwan; 3 Genomics Research Center, Academia Sinica, Taipei, 115 Taiwan; 4 Agricultural Biotechnology Research Center, Academia Sinica, Taipei, 115 Taiwan

## Abstract

Glucagon-like peptide-1 (GLP-1) receptor (GLP-1R) analogues are approved for treating type 2 diabetes, but are known to activate GLP-1R signaling globally and constitutively. Active compound N55, previously isolated from fenugreek, enhances the potency of GLP-1 without activating GLP-1R. Here we investigated if N55 lowers plasma glucose base on physiological levels of GLP-1. N55 was found to dose-dependently lower plasma glucose in non-fasted mice but not in the fasted mice, with the effect attenuated by GLP-1R antagonist exendin-(9–39) (Ex-9). On the other hand, when co-administered with dipeptidyl peptidase-IV (DPP4) -resistant [Aib8]-GLP-1(7–36) amide (GLP-1′), hypoglycemic response to N55 was observed in the fasted mice. This enhancement was also found to display dose dependency. N55 enhancement of the hypoglycemic and insulinotropic action of GLP-1′ was eliminated upon Ex-9 treatment. Both exendin-4 (Ex-4) and DPP4-resistant GLP-1 mutant peptide ([Aib8, E22, E30]-GLP-1(7–36) amide) activated GLP-1R and improved glucose tolerance but the enhancement effect of N55 was not observed *in vivo* or *in vitro*. In conclusions, N55 lowers plasma glucose according to prandial status by enhancing the response of physiological levels of GLP-1 and is much less likely to disrupt tight regulation of GLP-1R signaling as compare to GLP-1 analogues.

## Introduction

Glucagon-like peptide-1 (GLP-1) receptor (GLP-1R) is expressed in many peripheral and neuronal tissues and is activated by circulating GLP-1 or by neuron-secreted GLP-1. The endogenous active form of GLP-1 (GLP-1 (7–36) amide) rapidly rises 3–4 fold postprandially to maintain normoglycemia^1,2^. In general, active GLP-1 is quickly secreted after food intake and rapidly cleared by dipeptidyl peptidase-IV (DPP4) and the kidneys^3^. The almost ubiquitous presence of the GLP-1R contrasts with the extremely short half-life of the peptide in the circulation, implying that GLP-1 is tightly and spatiotemporally regulated in healthy subjects.

GLP-1 analogues have been approved for treatment of type 2 diabetes^4^. However, these analogues initiate a global and chronic activation of GLP-1Rs whose functions may be totally unrelated to their therapeutic indication. The target tissues and physiological message transmission pathways of the pharmacological level of GLP-1 analogues may be quite different from those of physiological levels of GLP-1^2,5,6^. All these features disrupt the tight regulation of GLP-1R signaling and may ultimately lead to adverse effects^7^.

To avoid the un-physiological features associated with the current GLP-1 analogue therapies, we have isolated an active compound (N55) from fenugreek seeds that sets off the signaling pathway of GLP-1R without directly activating GLP-1R^8^. N55 binds and enhances the potency of GLP-1 to stimulate GLP-1R *in vitro*, but does not enhance the potency of gastric inhibitory peptide (GIP), glucagon or exendin-4 (Ex-4), suggesting that N55 selectively interacts with GLP-1 peptide^8^.

To explore whether N55 is able to improve glucose tolerance without disrupting tight regulation of GLP-1R, we analyzed the responses of N55 in animal studies. Since the acute responses from manipulating GLP-1 activity is reflected by changes in plasma glucose, glucose excursions were measured to monitor the response of N55 in animal studies. Two key concerns were addressed. Firstly, whether N55 could lower plasma glucose according to the physiological need. Secondly, whether the hypoglycemic effect of N55 was specifically GLP-1 dependent. As endogenous GLP-1 levels were found to be high in non-fasted mice and were reduced to minimum after the mice were fasted. Glycemic responses of N55 in non-fasted mice and in fasted mice were used to address these concerns. DPP4 resistant [Aib8]-GLP-1(7–36) amide (GLP-1′), DPP4-resistant GLP-1 mutant peptide ([Aib8, E22, E30]-GLP-1(7–36) amide) and Ex-4 were used to explore mechanism of action in animal studies. Examining the effect of N55 on hypoglycemic response of GLP-1′, Ex-4 and [Aib8, E22, E30]-GLP-1(7–36) amide allowed us to specify the primary target of N55 *in vivo*.

## Results

### N55 dose dependently lowered plasma glucose in non-fasted mice but not in fasted mice

**Figure 1:**
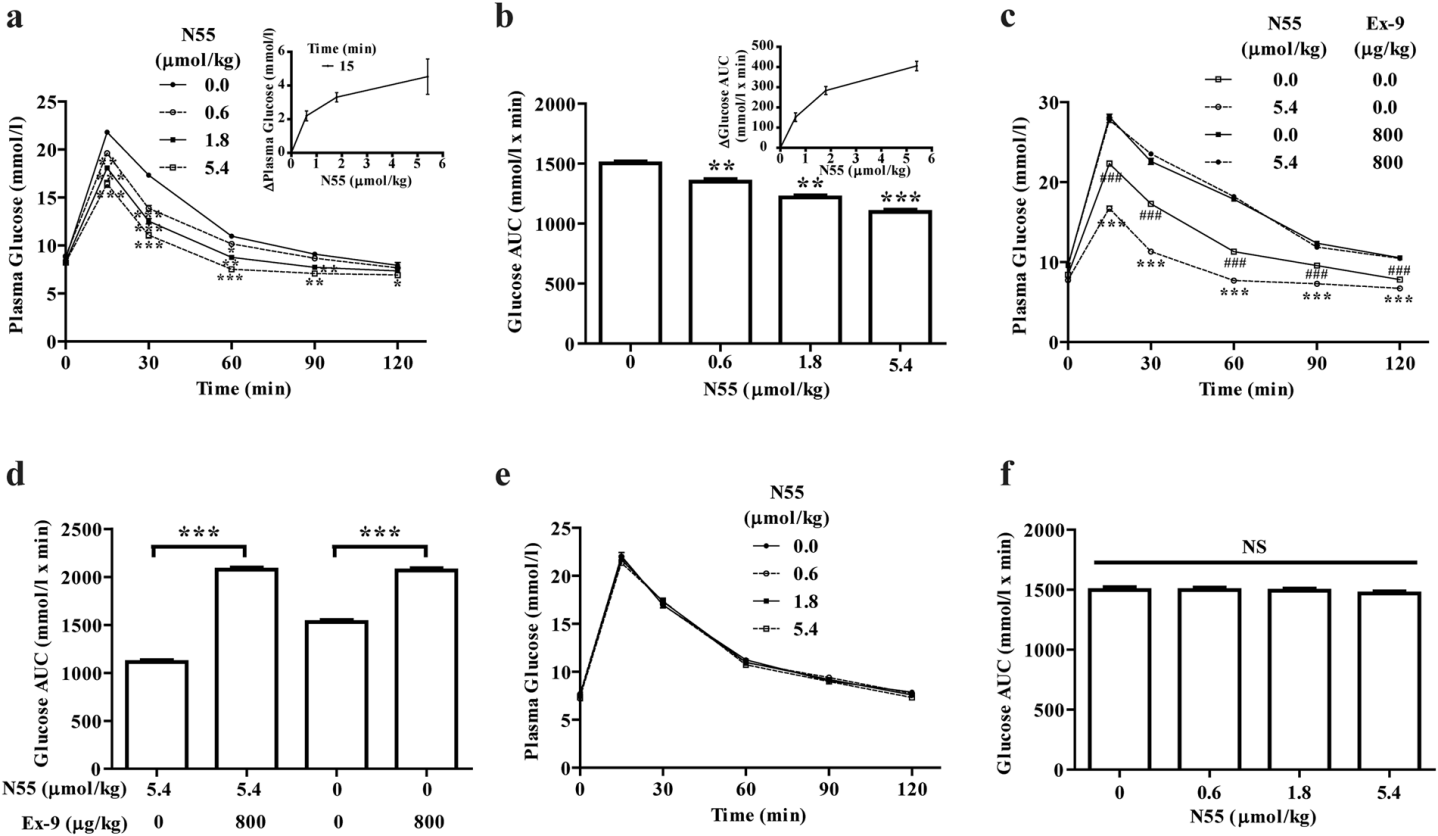
N55 improved glucose tolerance of IPGTT in non-fasted but not in fasted mice. Effect of N55 on (**a**) plasma glucose levels of non-fasted mice (inset shows the dependence of decrease in plasma glucose on N55) and on (**b**) glucose AUC_0–120_ of non-fasted mice [inset shows the dependence of decrease in glucose AUC_0–120_ on N55]. Effect of Ex-9 and N55 on (**c**) plasma glucose levels of non-fasted mice and on (**d**) glucose AUC_0–120_ of non-fasted mice. Effect of N55 on (**e**) plasma glucose and on (**f**) glucose AUC_0–120_ of 4 h-fasted mice. Indicated doses of N55 or Ex-9 were i.p. administered to group of mice (n = 5)15 min before glucose loading (2 g/kg). Values are mean ± SEM for groups of five mice. * *P* < 0.05, ** *P* < 0.01 and *** *P* < 0.001 were comparisons of indicated dose of N55 vs control (**a**,**b**). *** *P* < 0.001 and ^**###**^ *P* < 0.001 were comparisons of indicated dose of N55 in the absence vs presence of Ex-9 (**c**,**d**). NS, not significant.

To test if N55 would affect the plasma glucose, we examined glycemic response of N55 during intraperitoneal glucose tolerance test (IPGTT) in non-fasted mice. As shown in Fig. 1a, non-fasted mice were intraperitoneal (i.p.) administered with 0.6, 1.8 or 5.4 μmol/kg of N55, in which all three doses markedly reduced the plasma glucose. The most pronounced effect was found 15 min after i.p. glucose loading (Fig. 1a), the glucose levels at 15 min time points were lowered by N55 in a dose-dependent manner (Inset of Fig. 1a). The hypoglycemic response declines sharply at 60 min point. This observation is consistent with the plasma level of N55 during the first 60 min of IPGTT (Supplementary Method and Fig. S1). The maximal plasma level of N55 was 40.71 nmol/l 5 min after i.p. administering 1.8 μmol/kg of N55. The plasma concentration was rapidly decreased to 23 and 7.46 nmol/l 30 and 75 min after administration, respectively. Time points of 5, 30 and 75 min were chosen to measure the plasma level of N55 because they corresponds to −10, 15 and 60 min time points in the IPGTT study. As the hypoglycemic response was rapidly declined 60 min after glucose loading, the plasma level of N55 was also sharply decline 75 min after its administration. The improvement of glucose excursion as measured by the calculated area under the glucose concentration curve (glucose AUC_0–120_) was quantitatively and positively correlated with the dose of N55 ranging from 0.6 to 5.4 μmol/kg (Fig. 1b and Inset). The result demonstrated that N55 dose-dependently improved glucose tolerance in non-fasted mice. The hypoglycemic action of N55 was completely eliminated by GLP-1R antagonist exendin-(9–39) (Ex-9) (Fig. 1c). The glucose AUC_0–120_ was decreased from 1530 to 1113 mmol/l x min glucose by N55 but were both increased to 2070 mmol/l x min glucose by Ex-9 (Fig. 1d). The contribution of endogenous GLP-1 to the improvement of glucose excursion in non-fasted mice can be illustrated by the difference between the glucose AUC_0–120_ in the absence and presence of Ex-9. This value (by subtracting glucose AUC_0–120_ in the absence of Ex-9 from that in the presence of Ex-9) was calculated to be 537 mmol/l x min glucose and was further increased by N55 to 963 mmol/l x min glucose. To test the involvement of endogenous GLP-1, we examined the effect of N55 on plasma glucose levels in fasted mice whose physiological levels of endogenous GLP-1 were substantially reduced. Administering N55 from 0 to 5.4 μmol/kg did not affect the plasma glucose levels in the fasted-mice (Fig. 1e) and did not affect glucose excursions (Fig. 1f).

### Improvement of glucose tolerance by N55 required the presence of GLP-1′

**Figure 2:**
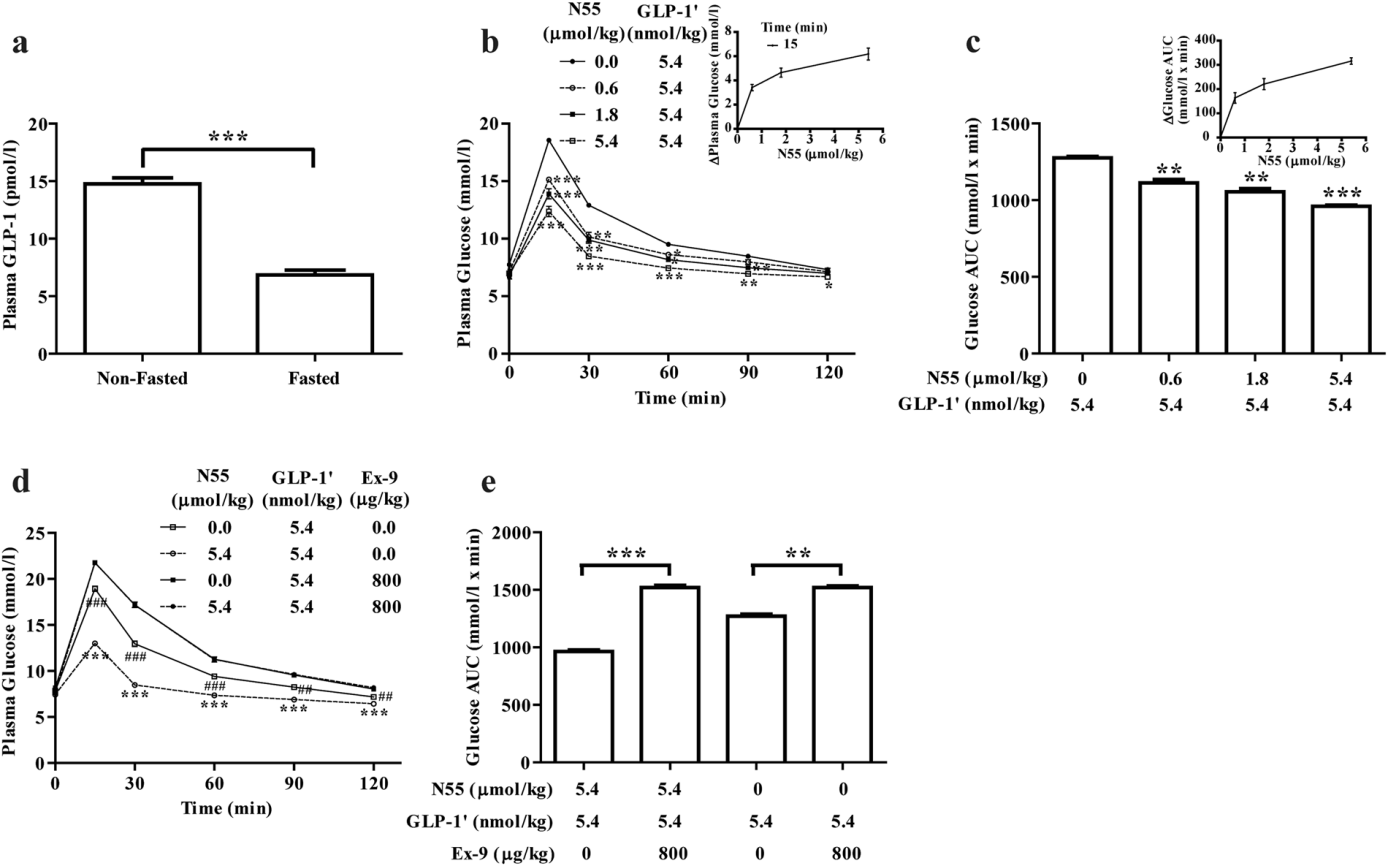
N55 improved glucose tolerance during IPGTT of fasted mice co-administered with GLP-1′. (**a**) Plasma GLP-1 level in non-fasted and fasted mice. Dose effect of N55 on (**b**) plasma glucose levels [inset shows the dependence of decrease in plasma glucose on N55] and on (**c**) glucose AUC_0–120_ [inset shows dependence of decrease in glucose AUC_0–120_ on N55] of 5.4 nmol/kg of GLP-1′. Effect of Ex-9 on GLP-1′ elicited (**d**) plasma glucose levels and (**e**) glucose AUC_0–120_ in the presence or absence of N55. Indicated doses of N55, GLP-1**′** or Ex-9 were i.p. administered to group of fasted mice (n = 5)15 min before glucose loading (2 g/kg). Values are mean ± SEM for groups of five mice. * *P* < 0.05, ** *P* < 0.01 and *** *P* < 0.001 were comparisons of non-fasted vs fasted mice (**a**), and were comparisons of 5.4 nmol/kg of GLP-1′ in the presence of indicated dose of N55 vs GLP-1′ alone (**b**,**c**). ** *P* < 0.01, *** *P* < 0.001, ^**##**^ *P* < 0.01 and ^**###**^ *P* < 0.001 were comparisons of indicated dose of N55 and 5.4 nmol/kg of GLP-1′ in the absence vs presence of Ex-9 (**d**,**e**). NS, not significant.

Since GLP-1 levels are substantially reduced in fasted mice^9^ (Fig. 2a), we restored GLP-1 levels in fasted mice by administering a DPP4 resistant version of GLP-1 (GLP-1′)^10^, and then examined glycemic responses of N55. Figure 2b showed that N55 dose-dependently lowered plasma glucose during IPGTT of fasted mice co-administered with a fixed dose of GLP-1′ (5.4 nmol/kg). 5.4 nmol/kg of GLP-1′ significantly improved the glucose tolerance (Figs 1e, 2b) and glucose excursion (Figs 1f, 2c) in fasted mice. The 15-min plasma glucose level was further decreased from 18 mmol/l to 15, 14 or 12 mmol/l after co-administering 0.6, 1.8 or 5.4 μmol/kg of N55, respectively. N55 dose-dependently decreased plasma glucose (inset of Fig. 2b) and glucose AUC_0–120_ (inset of Fig. 2c) of GLP-1′. The N55 enhanced glycemic responses were pronounced at 15 min time point and declined rapidly at 60 min time point (Fig. 2b). The glucose AUC_0–120_ of 5.4 nmol/kg of GLP-1′ was also reduced as the N55 increased from 0.6 to 5.4 μmol/kg (Fig. 2c).

Co-administering the mice with Ex-9 completely eliminated the glucose lowering effect of GLP-1′ and the enhancement effect of N55, in which the glucose levels were increased to 22 mmol/l (Fig. 2d) and the glucose AUCs had a value close to 1520 mmol/l x min glucose (Fig. 2e) in all the Ex-9 treated mice. The contribution of 5.4 nmol/kg of GLP-1′ to the improvement of glucose excursion was measured to be 250 mmol/l x min glucose (by subtracting 1270 mmol/l x min glucose from 1520 mmol/l x min glucose). This value was increased to 560 mmol/l x min glucose with N55 (subtracting 960 mmol/l x min glucose from 1520 mmol/l x min glucose), showing N55 enhanced the improvement of glucose excursion of GLP-1′. Both of these values were reduced to non-detectable level by Ex-9. This analysis showed that the enhancement effect of N55 depended on the GLP-1R signaling.

### Enhancement effect of N55 depended on the dose of GLP-1′

**Figure 3:**
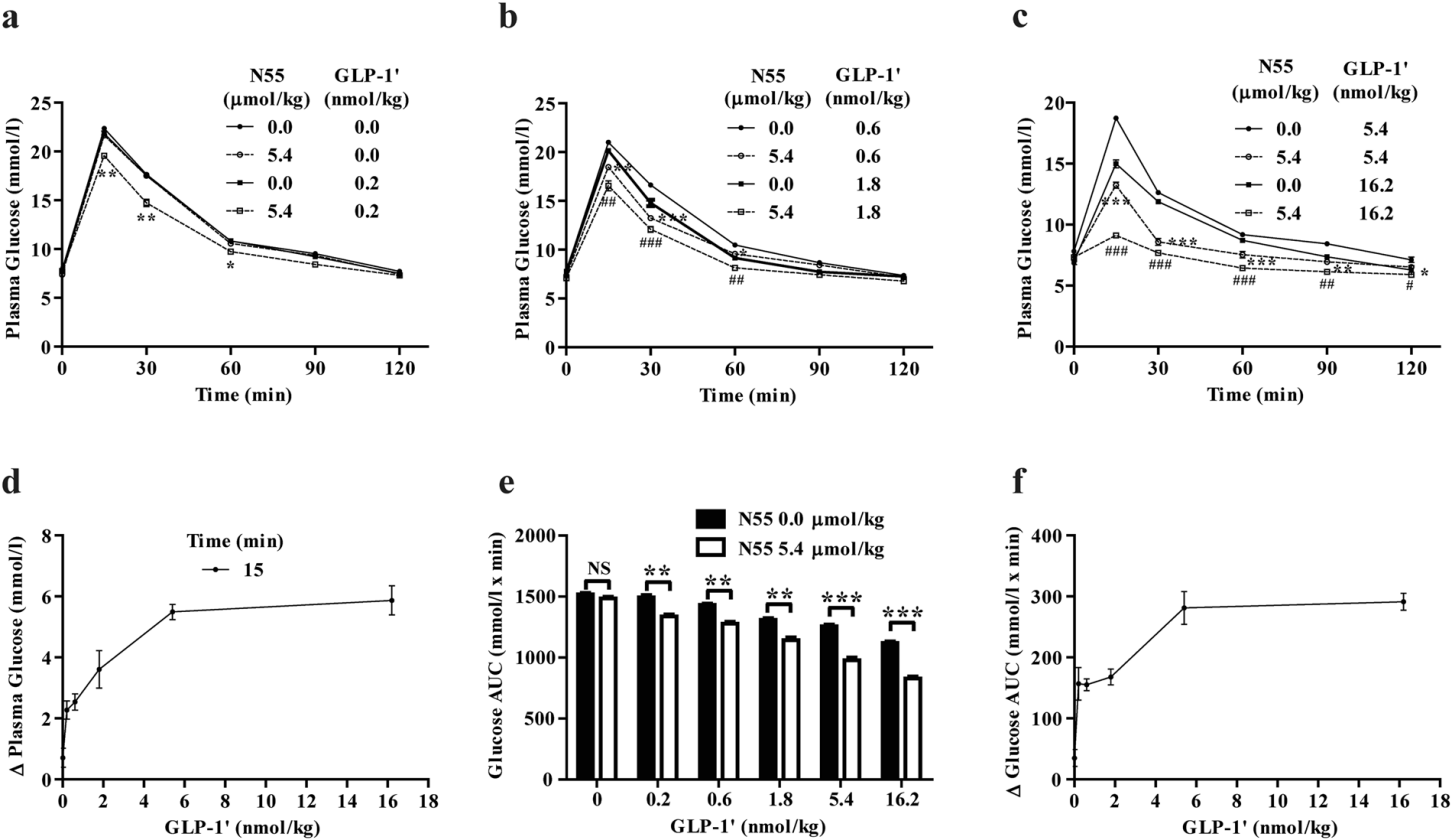
Hypoglycemic effect of N55 depended on the dose of GLP-1′ in fasted mice. Effect of N55 (5.4 μmol/kg) on plasma glucose levels in the presence of (**a**) 0 or 0.2, (**b**) 0.6 or 1.8, and (**c**) 5.4 or 16.2 nmol/kg of GLP-1′. (**d**) Dependence of decrease in plasma glucose by N55 and dose of GLP-1′ at the 15 min time points. (**e**) Effect of N55 on glucose AUC_0–120_ of indicated dose of GLP-1′. (**f**) Dependence of decrease in glucose AUC_0–120_ by N55 and dose of GLP-1′. Indicated doses of N55 and GLP-1**′** were i.p. administered to group of fasted mice (n = 5)15 min before glucose loading (2 g/kg) of the IPGTT. Values are mean ± SEM for groups of five mice. * *P* < 0.05, ** *P* < 0.01, *** *P* < 0.001, ^**#**^ *P* < 0.05, ^**##**^ *P* < 0.01 and ^**###**^ *P* < 0.001 were comparisons of indicated dose of GLP-1′ in the presence vs absence of N55. NS, not significant.

To examine if the N55-enhanced hypoglycemic responses were dependent on the dose of GLP-1′, we administered the fasted mice with increasing doses of GLP-1′ together with a fixed dose of N55 (5.4 μmol/kg) followed by IPGTT. Administering 0.2 nmol/kg of GLP-1′ alone did not affect the glucose tolerance at all (Fig. 3a). However, this barely detectable response was significantly enhanced by N55 (Fig. 3a). As the dose of GLP-1′ was increased to 0.6 through to 16.2 nmol/kg, the enhancement of improved glucose tolerance by N55 became more and more pronounced (Fig. 3b,c). The enhanced decrease in glucose levels of each dose of GLP-1′ by N55 at 15 min time points was dependent on the dose of GLP-1′ and became saturated as the dose reached 5.4 nmol/kg (Fig. 3d). Comparing the effect of N55 on glucose AUCs of each indicated dose of GLP-1′ revealed that glucose AUC_0–120_ was further decreased by N55 (Fig. 3e). This enhancement effect of N55 on the decrease in glucose AUC_0–120_ was directly related to the dose of GLP-1′ and became saturated at a GLP-1′ dose of 5.4 nmol/kg (Fig. 3f).

### N55 enhanced the insulinotropic responses of GLP-1

**Figure 4:**
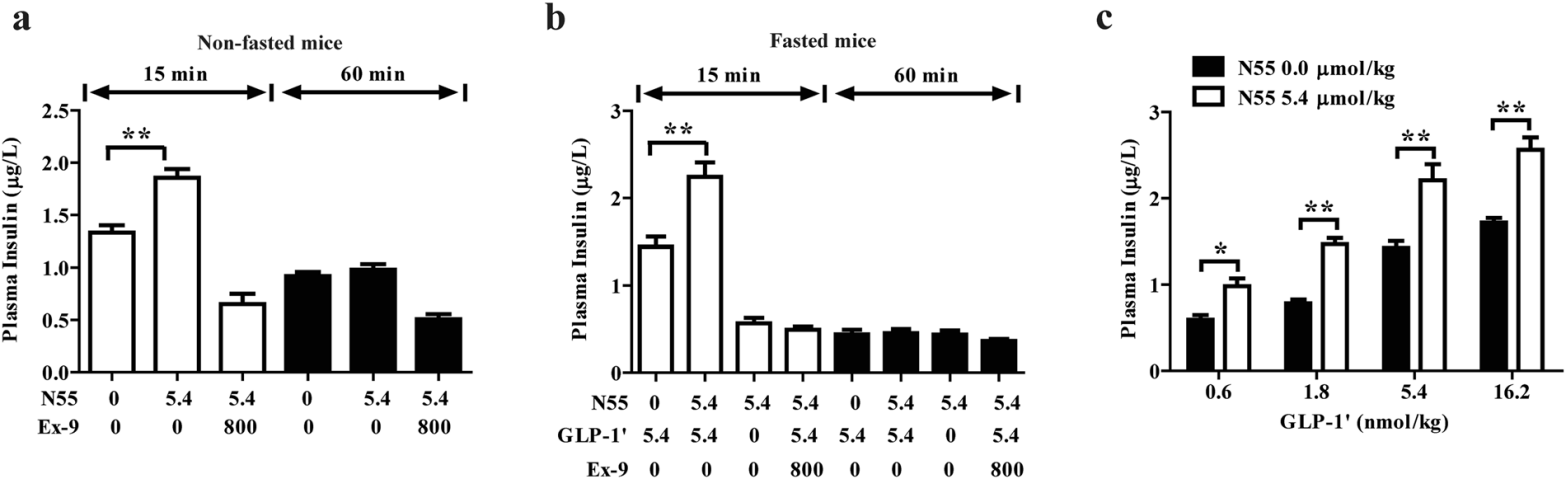
N55 increased insulinotropic effect of GLP-1 in mice IPGTT. Effect of N55 on plasma insulin levels 15 min and 60 min following an IPGTT glucose loading in (**a**) non-fasted mice and (**b**) fasted mice. (**c**) Effect of N55 and indicated dose of GLP-1′ on plasma insulin level 15 min after glucose loading. Indicated doses of N55, GLP-1′ and Ex-9 were i.p. administered alone or in combination to group of mice (n = 5)15 min before glucose loading (2 g/kg).Values are mean ± SEM for groups of five mice. * *P* < 0.05 and ** *P* < 0.01 were comparisons of indicated dose of GLP-1′ in the absence or presence of N55.

To assess whether N55 improves glucose tolerance by enhancing the insulinotropic effect of GLP-1, we compared the plasma insulin levels from mice with or without receiving N55 intraperitoneally 15 and 60 min after i.p. glucose loading. The 15-min plasma insulin levels from non-fasted mice were increased from 1.33 to 1.86 μg/L by N55 (Fig. 4a) and those of fasted mice receiving GLP-1′ were increased from 1.44 to 2.24 μg/L by N55 (Fig. 4b) While the effect of N55 on the insulin level at 60-min after glucose loading was markedly reduced, was in agreement with the sharp decline in its glycemic response (Figs 1a, 2b). Ex-9 reduced the levels of insulin to 0.36–0.65 μg/L 15 and 60 min after glucose loading (Fig. 4a,b). This demonstrated that N55 enhanced the insulinotropic effect of GLP-1 and the effect required the function of GLP-1R signaling. Furthermore, the enhancement of insulinotropic responses by a fixed dose of N55 were correlated to the doses of administered GLP-1′ (Fig. 4c), showing that higher level of GLP-1′ would result in the more pronounced effect of N55.

**Figure 5:**
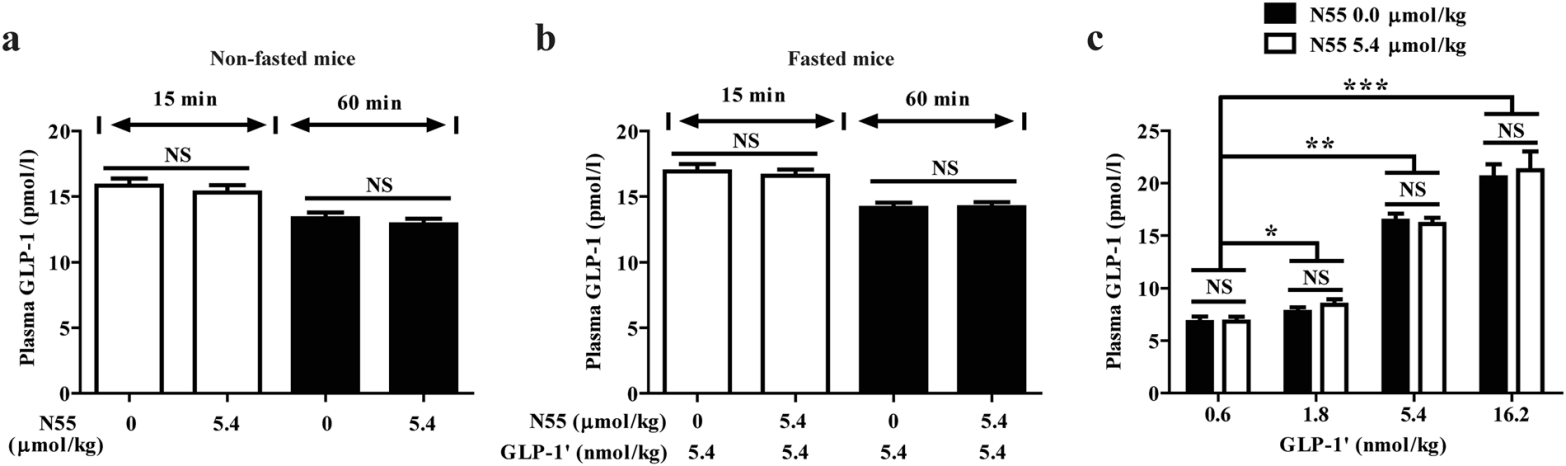
N55 did not affect plasma GLP-1 levels. Plasma GLP-1 levels were measured at 15 min and 60 min following glucose loading in (**a**) non-fasted mice and (**b**) fasted mice treated with indicated dose of N55 and GLP-1′. (**c**) Plasma GLP-1 levels 15 min after an i.p. glucose loading of fasted mice i.p. receiving indicated dose of GLP-1′ and N55. Indicated doses of N55 or GLP-1′ were alone or combined together i.p. administered to group of mice (n = 5)15 min before i.p. glucose loading (2 g/kg). Values are mean ± SEM for groups of five mice. * *P* < 0.05, ** *P* < 0.01 and *** *P* < 0.001 were comparisons of 1.8, 5.4 and 16.2 nmol/kg of GLP-1′ vs 0.6 nmol/kg of GLP-1′, and the comparisons in the presence vs absence of N55. NS, not significant.

To examine if the N55-enhanced hypoglycemic and insulinotropic responses of GLP-1 is due to enhancement of endogenous GLP-1 secretion, we measured the plasma GLP-1 levels 15 and 60 min after IPGTT glucose loading in mice with or without receiving N55. As shown in Fig. 5, N55 did not affect the levels of GLP-1 in non-fasted mice (Fig. 5a) nor in fasted mice receiving GLP-1′ or not (Fig. 5b,c). This finding is consistent with the observation that N55 will not act as a DPP4 inhibitor (Supplementary Method and Fig. S2).

### Hypoglycemic action of Ex-4 was not affected by N55

**Figure 6:**
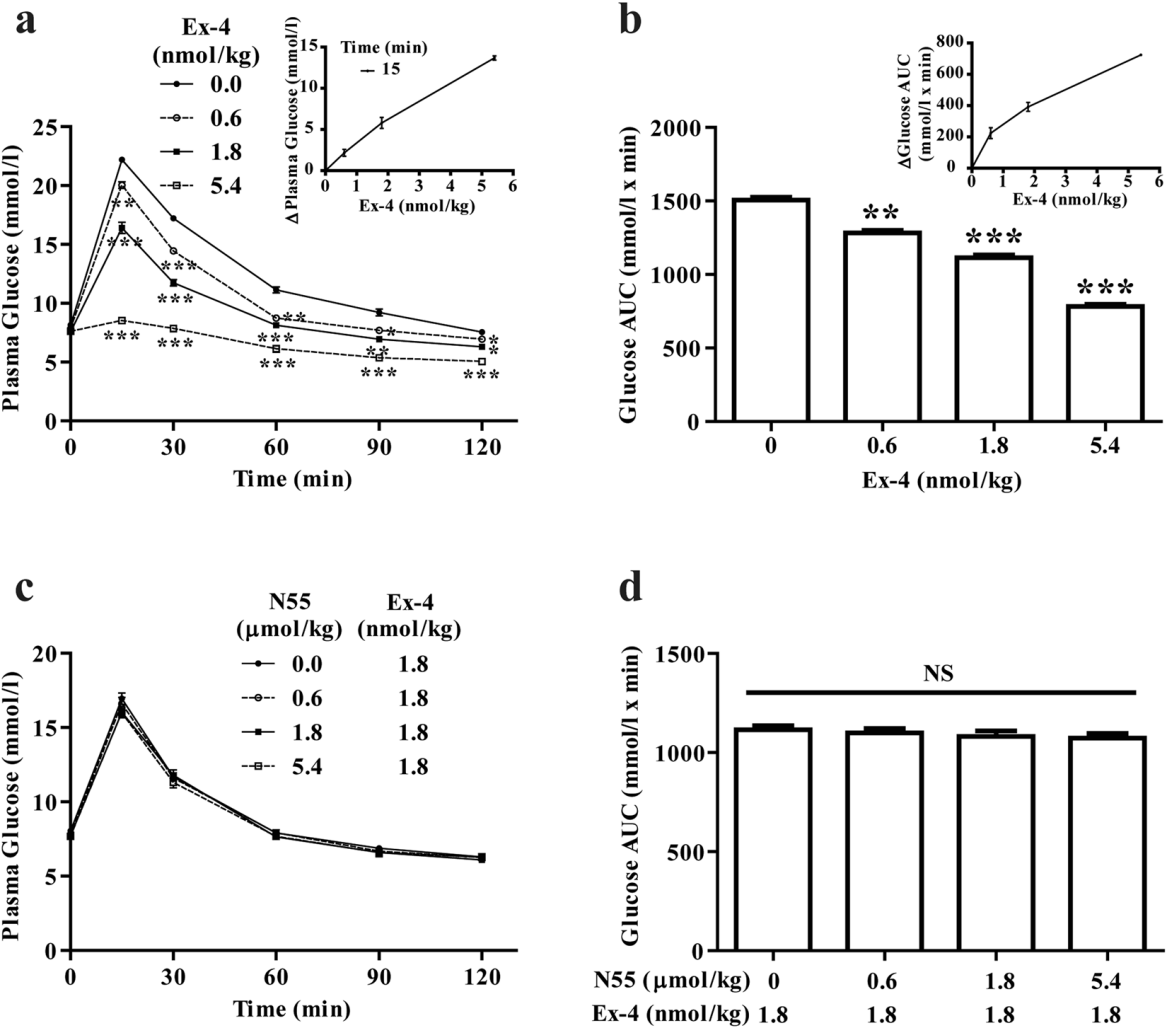
N55 did not affect glucose tolerance of Ex-4 in fasted mice. Dose effect of Ex-4 on (**a**) plasma glucose levels [inset shows dependence of decrease in plasma glucose on dose of Ex-4] and on (**b**) glucose AUC_0–120_ [Inset shows the dependence of decrease in glucose AUC_0–120_ on doses of Ex-4]. Dose effect of N55 on (**c**) plasma glucose levels and on (**d**) glucose AUC_0–120_ of 1.8 nmol/kg of Ex-4. Indicated doses of Ex-4 and N55 were i.p. administered to group of fasted mice (n = 5)15 min before the glucose loading (2 g/kg) of the IPGTT. Values are mean ± SEM for groups of five mice. * *P* < 0.05, ** *P* < 0.01 and *** *P* < 0.001 were comparisons of indicated dose of Ex-4 vs control (**a**,**b**). NS, not significant.

All the observations so far indicated that N55 enhanced the glycemic control of GLP-1 by enhancing its potency. We examined whether the hypoglycemic effect of Ex-4 (a potent GLP-1 mimetic) can be enhanced by N55 while N55 does not affect the potency of Ex-4 *in vitro* ^8^. Administering Ex-4 to fasted mice greatly improved the glucose tolerance (Fig. 6a). The decrease in glucose level depended on the dose of Ex-4 (Inset of Fig. 6a). The glucose AUCs were also decreased as dose of Ex-4 increased (Fig. 6b). The glucose AUC_0–120_ was decreased from 1500 to 780 mmol/l x min glucose as the dose of Ex-4 was increased to 5.4 nmol/kg (Fig. 6b) and the decrease depended on dose of Ex-4 (Inset of Fig. 6b). While Ex-4 potently reduced plasma glucose and improved glucose excursion, its hypoglycemic effect and glucose AUCs were not affected by dose of N55 from 0.6 to 5.4 μmol/kg (Fig. 6c,d). These results illustrated that N55 did not affect the glucose tolerance of Ex-4.

### N55 did not enhance the hypoglycemic action of [Aib8, E22, E30]-GLP-1(7–36) amide

**Figure 7:**
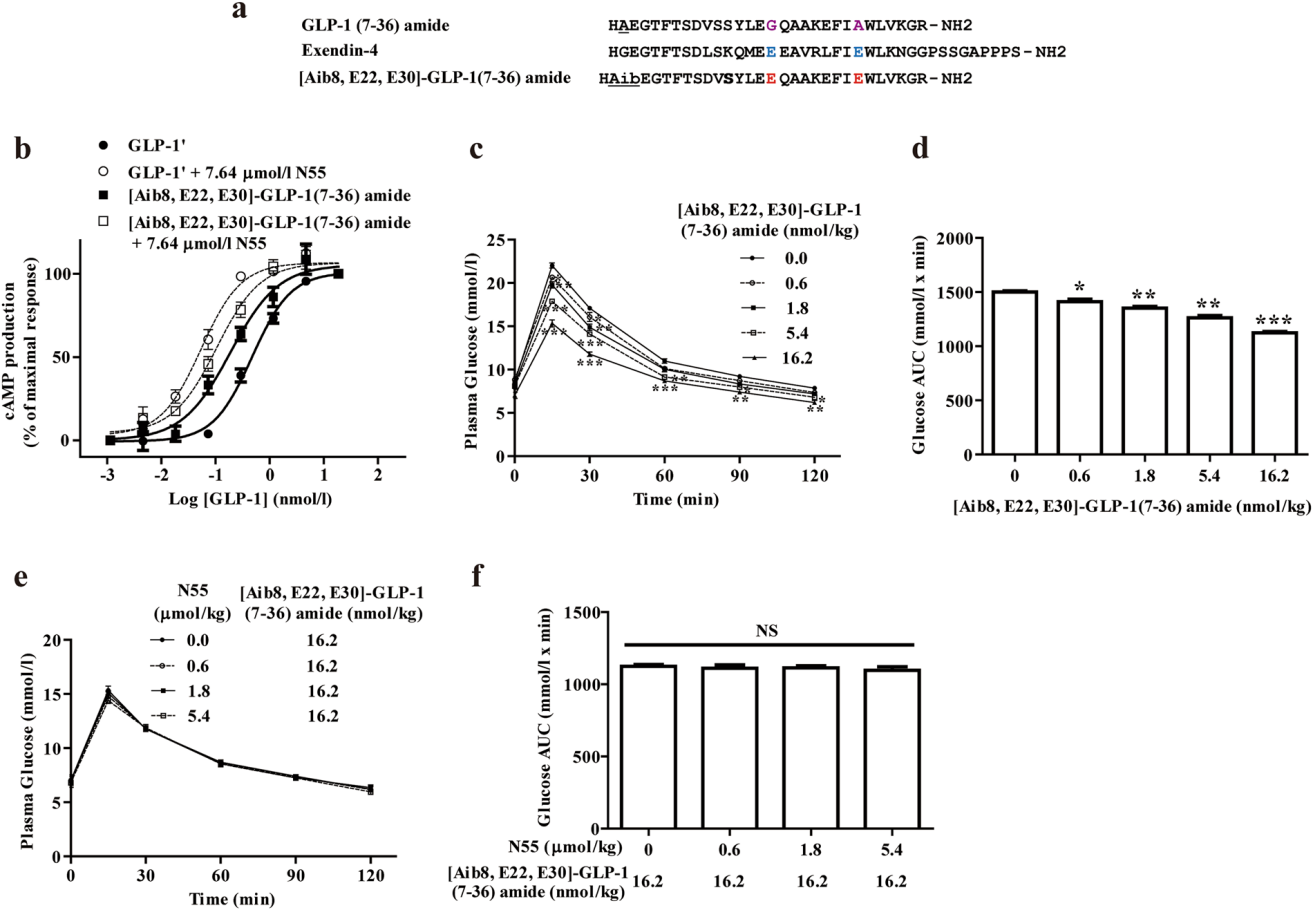
Hypoglycemic action of [Aib8, E22, E30]-GLP-1(7–36) amide was not affected by N55. (**a**) Sequence alignment of GLP-1(7–36) amide, Ex-4 and mutant peptide. G22 and A30 (purple alphabets) are non-homologous residues between GLP-1(7–36) amide and Ex-4. These residues in GLP-1(7–36) amide were replaced with E (red alphabets), the corresponding residues (blue alphabets) in Ex-4, to generate DPP4-resistant GLP-1 mutant peptide ([Aib8, E22, E30]-GLP-1(7–36) amide). Effect of N55 on cAMP responses to the titration of (**b**) [Aib8, E22, E30]-GLP-1(7–36) amide and GLP-1′. Values are means ± SEM of triplicate assays from three independent experiments. Dose effect of [Aib8, E22, E30]-GLP-1(7–36) amide on (**c**) plasma glucose levels and on (**d**) glucose AUC_0–120_. Dose effect of N55 on (**e**) the hypoglycemic response and on (**f**) glucose AUC_0–120_ of 16.2 nmol/kg of [Aib8, E22, E30]-GLP-1(7–36) amide. Indicated doses of [Aib8, E22, E30]-GLP-1(7–36) amide and N55 were i.p. administered to group of fasted mice (n = 5) 15 min before the glucose loading (2 g/kg) of the IPGTT. Values are mean ± SEM for groups of five mice. * *P* < 0.05, ** *P* < 0.01 and *** *P* < 0.001 were comparisons of indicated dose of [Aib8, E22, E30]-GLP-1(7–36) amide vs control (**c**,**d**). NS, not significant.

A point mutant of GLP-1 was constructed, which was not responsive to the enhancement of N55 *in vitro* but retained ability to activate the receptor. Since Ex-4 is not responsive to N55, we replaced residues in GLP-1 with the corresponding residues in Ex-4 to generate [E22, E30] GLP-1 (7–36) amide. DPP4-resistant GLP-1 mutant peptide ([Aib8, E22, E30]-GLP-1(7–36) amide) was constructed by replacing Ala 8 with aminoisobutyric acid (Aib) (Fig. 7a). The DPP4 resistant version [Aib8, E22, E30]-GLP-1(7–36) amide displayed comparable potency in stimulating GLP-1R, but was failed to respond to N55 (Fig. 7b). [Aib8, E22, E30]-GLP-1(7–36) amide dose-dependently lowered the plasma glucose (Fig. 7c) and decreased glucose AUCs (Fig. 7d) during IPGTT in fasted mice. However, the hypoglycemic effect (Fig. 7e) and glucose AUC_0–120_ (Fig. 7f) of [Aib8, E22, E30]-GLP-1(7–36) amide were not affected by N55 at all. These studies showed that replacing glycine (G) 22 and alanine (A) 30 of native GLP-1 with the corresponding glutamate (E) in Ex-4 led to loss of the enhancement response to N55.

## Discussion

The primary aim of the present study is to test if N55 can modulate GLP-1 activity *in vivo*. To minimize the potential involvement of endogenous incretins from enteroendocrine cells on plasma glucose level, DPP-4 resistant GLP-1 (GLP-1′)^10^, Ex-4 and N55 were all i.p. administered 15 min before IPGTT study. Food intake will stimulate incretins secretion and raise their plasma levels, while fasting will lead to a reduction in plasma incretin levels^11^. Our present finding showed that plasma GLP-1 level is much lower in fasted mice (Fig. 2a) and is consistent with findings from other independent studies. N55 dose-dependently improved glucose tolerance in non-fasted mice but not in fasted mice (Fig. 1), showing an effect depended on the prandial status and GLP-1 levels. Analysis of glucose AUC_0–120_ by Ex-9 revealed that physiological levels of GLP-1 contributed significantly to the glycemic control in non-fasted mice and its contribution was further enhanced by N55 (Fig. 1d). Conversely, the level of the endogenous GLP-1 in fasted mice is much lower (Fig. 2a) and its contribution to glycemic control was not detected and was not affected by N55 (Fig. 1e,f). GLP-1 is required for glycemic control postprandially while it becomes superfluous in the fasting status^9^. This correlated with the action of N55 in non-fasted and fasted mice. Increased dose of N55 up to 5.4 μmol/kg was not able to affect the response of endogenous GLP-1 level in the fasted mice. Thus the present result illustrated that the hypoglycemic action of N55 was operated according to physiological requirements and was mediated by physiological levels of GLP-1.

N55 was metabolized moderately in blood stream, the maximal level was 40.71 nmol/l 5 min after its administration and was declined sharply 75 min later. This kinetic analysis is consistent with the time frame of N55’s action in non-fasted mice and GLP-1′-treated fasted mice. Hypoglycemic effects (Figs 1a, 2b) and insulinotropic effects (Fig. 4a,b) were sharply reduced at 60 min time point of IPGTT studies. This finding may indicate that the intact N55 is required to enhance the biological responses *in vivo*.

Several lines of evidence indicated that the hypoglycemic action of N55 was due to its effect on GLP-1 peptide. Firstly, the hypoglycemic effect of N55 during IPGTT of non-fasted mice requires functional GLP-1R signaling. Secondly, although N55 could not affect the plasma glucose in fasted mice whose endogenous GLP-1 are relatively low (Fig. 2a). N55 dose-dependently improved glucose tolerance after the administration of GLP-1′ and the effect of N55 was dependent on the dose of GLP-1′. Thirdly, the hypoglycemic effect and potentiation of insulinotropic effect of GLP-1 required the functional signaling of GLP-1R. Finally, either Ex-4 or [Aib8, E22, E30]-GLP-1(7–36) amide displayed hypoglycemic action *in vivo* and stimulated GLP-1R *in vitro*. But N55 failed to enhance the potency of Ex-4 and [Aib8, E22, E30]-GLP-1(7–36) amide *in vitro* and did not affect their hypoglycemic responses in mice. In addition to islets, GLP-1 regulates glucose metabolism via neural networks consisting of GLP-1R expressing tissues and downstream tissues responsible for the message transmission and glucose production and disposal^9,12–16^. Since N55 did not affect the hypoglycemic action of Ex-4 or [Aib8, E22, E30]-GLP-1(7–36) amide, thus excluded the possibility that N55 acted directly on either GLP-1R or the downstream tissues. Furthermore, N55 did not affect endogenous GLP-1 levels and showed stringent specificity to enhance the response of native GLP-1. Together with previous *in vitro* characterization of N55^8^, it is most likely that the potency of GLP-1 is enhanced by N55 in mice. Thus the simplest interpretation is that N55 bound GLP-1 and enhanced its activity to stimulate GLP-1R *in vivo*, and consequently led to reduction in plasma glucose.

Plasma level of active GLP-1 is relatively low with a very short half-life^3^. This rapid metabolism of GLP-1 raises questions about how its hypoglycemic effects are mediated on target organs other than pancreatic beta cells. Indeed, more and more evidences indicate that physiological message transmission pathways and primary target tissues are found extrapancreatically^2,5,12,17,18^. DPP4 inhibitor (DPP4i) study^19^ and neurophysiological analyses^2,5,12,17,18^ indicate the GLP-1R expressing tissues around the hepatoportal area and enteric neurons are the primary target tissues for physiological levels of GLP-1 to lower plasma glucose. Conversely, the pancreatic beta cells are the primary target tissue for the hypoglycemic responses of pharmacological levels of GLP-1^2,5,6,12,17,18^. N55 enhanced the hypoglycemic response of physiological levels of GLP-1 and implied that the compound may target at the GLP-1R expressing neurons in gastrointestinal or hepatoportal areas. However, the exact target tissues still need further investigations. In attempts to develop orally available GLP-1R modulators, a diverse array of allosteric non-peptide ligands with intrinsic efficacy has been reported^20^. The reported GLP-1R positive modulators will bind the receptor at allosteric site with intrinsic agonistic activity^21,22^ and potentially lead to activation of GLP-1R globally and chronically. In contrast N55 did not activate the receptor and thus should not activate GLP-1R chronically and globally.

As the hypoglycemic response of N55 depended on the level of GLP-1, implying that its potential therapeutic effect could comply with the physiological need of GLP-1 and was less likely to disrupt the tight regulation of GLP-1R signaling. N55 is the first compound of a new class of modulators that enhance glucose tolerance of GLP-1 according to the physiological need, without activating GLP-1R globally and constitutively.

In addition to its anti-diabetic remedy^23,24^, extracts of fenugreek seeds also display neuroprotective properties^25^, beneficial effect on Parkinson’s disease^26,27^ and anti-inflammatory effects^28,29^ in disease animal models. The action of N55 is consistent with the current trials of GLP-1 analogues in treatment of psoriasis^30^, Alzheimer’s disease^31^ and Parkinson’s disease^32^. It will be interesting to test whether N55 will play a role in ameliorating these disorders in disease animal models.

Food stimulation of enteroendocrine L cells induces the release of GLP-1. The vagus nerve innervates visceral organs and have been shown to contribute primarily to the mediation of gut-derived GLP-1’s effects on insulin secretion and glycemic control^17,33^. The mode of gut-derived endogenous GLP-1 to regulate insulin secretion is quite distinct from that of exogenous GLP-1 analogues which activate pancreatic cells’ GLP-1R at pharmacological dose. Though we assayed and compared the plasma level of GLP-1 in non-fasted mice to those in fasted mice administered GLP-1′, their mode action and pharmacological consequence may not be the same. The present study is to test if N55 can modulate GLP-1 *in vivo* and the results are consistent with the hypothesis. However, it is warranted that further studies are needed to investigate the detail mechanism of N55’ action to modulate endogenous GLP-1 in non-fasted mice. Mice deficient in GLP-1R signaling are viable and do not display developmental defects, but the sequence of mammalian GLP-1 is invariant. Hence, the physiological functions of endogenous GLP-1 are not limited to glucose control, it is also involved in a number of brain responses and survival of mammal in the wilderness.

In summary, the present study presented a novel mechanism of action to modulate GLP-1R signaling *in vivo*. N55 improved glucose tolerance by enhancing the glycemic control of physiological levels of GLP-1 and was in compliance with the physiological need. N55 is a new class of compound that may provide a new approach for future discovery of novel therapeutics aiming at modulating G protein-coupled receptor (GPCR) signaling. However, further chronic repeat dosage studies of N55 are needed to evaluate if this compound can circumvent the un-physiological features of current GLP-1 analogues. Though N55 enhanced plasma insulin level in a GLP-1R dependent manner, we do not know whether it was directly from the stimulation of GLP-1R in islets or indirectly through neural networks^9,12–16^. Further we cannot exclude the possibility that insulin independent mechanisms^16,19,33–37^ are involved in the responses of N55. Detailed characterizations of the primary target tissues and physiological signal transmission pathway will be undertaken for future study.

## Methods

### Animals

All animal procedures were approved by the Institute of Biomedical Sciences Animal Care and Use Committee (Academia Sinica, Taiwan). The Experimental Animal Committee, Academia Sinica, Taiwan approved all animal experimental procedures. All animals were carefully looked after to ensure that their welfare were well looked after. Additionally, all the experimental procedures and the reagents used in this manuscript have been approved by The Ethic Committee, Academia Sinica. All methods were performed in accordance with the relevant guidelines and regulations by the Institute of Biomedical Sciences Animal Care and Use Committee (Academia Sinica, Taiwan) and The Ethic Committee (Academia Sinica, Taiwan). Female C57BL/6 mice (BioLAS CO, Yi-Lan Breeding Center, Taiwan) aged 8–12 weeks were housed on a 12-h light/12-h dark cycle and provided with food and water *ad libitum*. The mice were fed with the standard chow (PicoLab Rodent Diet 5053, LabDiet, St. Louis, MO, USA) diet which consisted of 13.2% fat, 24.7% protein, and 62.1% carbohydrates as a source of calories.

### Reagents

Ex-9 and Ex-4 was synthesized by Genomics BioSci & Technology (Taipei, Taiwan). [Aib8] GLP-1 (7–36) amide is the DPP4-resistant GLP-1 peptide (termed GLP-1′) with the sequence HAibEGTFTSDVSSYLEGQAAKEFIAWLVKGR-NH2 (where Aib stands for aminoisobutyric acid)^10^, and the [Aib8, E22, E30]-GLP-1(7–36) amide is the DPP4-resistant GLP-1 mutant peptide with the sequence HAibEGTFTSDVSSYLEEQAAKEFIEWLVKGR-NH2. Both of the two amides were synthesized by LifeTein (New Jersey, USA). Isotonic sodium chloride solution (0.9% sodium chloride) was purchased from Sintong Taiwan Biotech (Taoyuan, Taiwan). The RPMI-1640 tissue culture medium, minimum essential medium (MEM), phenol red-free MEM, HEPES, sodium pyruvate, fetal bovine serum (FBS), penicillin-streptomycin, L-glutamine, amphotericin B, gentamicin, and 0.05% trypsin-EDTA were purchased from Life Technologies (Carlsbad, CA, USA). Puromycin, G418 and D-(+)-glucose (G8270) were purchased from Sigma-Aldrich (St. Louis, MO, USA). Coelenterazine 400 a (DeepBlueC, C-320–1) was purchased from Gold Biotechnology (St. Louis, MO, USA). N55 was obtained as described previously^8^.

### Intraperitoneal glucose tolerance test (IPGTT)

All experiments were performed at approximately 1 PM. For the fasted mice, standard chow was removed away at 9 AM on the day of the IPGTT, but were provided water *ad libitum* for 4 h prior to the beginning of the test. All the administered materials were dissolved in isotonic sodium chloride solution (0.9% sodium chloride) containing 5% ethanol. Vehicle, N55, GLP-1′, [Aib8, E22, E30]-GLP-1(7–36) amide, Ex-4 or Ex-9 alone or in combination were administered by i.p. injection 15 min before D-(+)-glucose (2 g/kg of body weight) i.p. administration. Blood samples were drawn from the tail vein right before glucose loading (time 0) and at 15, 30, 60, 90 and 120 min after glucose administration. Plasma glucose was monitored using a glucometer (Accu-Check Performa, Roche, Basel, Switzerland). Glucose AUC_0–120_ was calculated during the 120-min time interval from IPGTT results. Glucose AUC_0–120_ was obtained using the formula AUC_0–120_ = [15 × (G_0_ + G_15_)/2] + [15 × (G_15_ + G_30_)/2] + [30 × (G_30_ + G_60_)/2] + [30 × (G_60_ + G_90_)/2] + [30 × (G_90_ + G_120_)/2] where G_0_, G_15_, G_30_, G_60_, G_90_ and G_120_ were blood glucose level at each time point during IPGTT. The decrease in plasma glucose was calculated by subtracting the plasma glucose in the presence of indicated reagent from that in the absence of indicated reagent; ∆ Plasma Glucose (mmol/l) = [plasma glucose at 15 min point obtained in the absence of indicated reagent] − [plasma glucose at 15 min point obtained in the presence of indicated reagent]. The decreased in glucose AUC_0–120_ was expressed by subtracting the AUC_0–120_ in the presence of f indicated reagent rom that in the absence of indicated reagent. ∆ AUC_0–120_ (mmol/l x min) = [AUC_0–120_ in the absence of indicated reagent] − [AUC_0–120_ in the presence of indicated reagent].

### Plasma levels of GLP-1 measurement

Blood samples were collected from the tail vein before any drug administration and at 15 and 60 min after i.p. glucose loading (time 0), as well as added DPP4i (10 μL per milliliter of blood) by following the manufacturer’s protocols. Plasma GLP-1 levels were determined by a high sensitivity GLP-1 active chemiluminescent 96-Well plate assay (EZGLPHS-35K, Merck Millipore).

### Plasma levels of insulin measurement

Blood samples were collected from the tail vein before any drug administration and at 15 and 60 min after i.p. glucose loading (time 0). Plasma was separated by centrifugation at 4 °C before being stored at −20 °C until assay. Plasma insulin levels were determined by a mouse insulin enzyme-linked immunosorbent assay kit (10–1247–01, Mercodia, Sweden) according to the manufacturer’s protocols.

### Bioluminescence resonance energy transfer (BRET) and cyclic adenosine 3′,5′- monophosphate (cAMP) response assay

The real-time intracellular cAMP assay was performed as previously described^8^.

### Statistical analysis

Values for each experimental group are presented as mean ± SEM. Statistics were performed using Student’s *t*-test and one-way analysis of variance (ANOVA). Significance levels shown in the figures are: * *P* < 0.05, ** *P* < 0.01 and *** *P* < 0.001 as well as ^**#**^ *P* < 0.05, ^**##**^ *P* < 0.01 and ^**###**^ *P* < 0.001. A P-value of less than 0.05 was considered significant. Student’s *t*-test that is used to compare the means of two groups and one-way ANOVA is used to compare the means of more than two groups. Five mice per group were used for the *in vivo* studies. The *in vivo* studies were performed at least twice. The *in vitro* assays were triplicates of three independent experiments.

## Electronic supplementary material

Supplementary Information
