# Supplementary material for: Fenugreek Compound (N55) Lowers Plasma Glucose through the Enhancement of Response of Physiological Glucagon-like peptide-1
Source: Sci Rep. 2017 Sep 25;7:12265. doi: 10.1038/s41598-017-12290-x (PMC5613011; doi:10.1038/s41598-017-12290-x)
Supplement: Supplementary file 1 — Supplementary Information [file 41598_2017_12290_MOESM1_ESM.pdf]

Supplementary Information

Fenugreek Compound (N55) Lowers Plasma Glucose through the Enhancement of Response of Physiological Glucagon-like peptide-1

I-Wen Chou[1,2], Yu-Hong Cheng[3], Yet-Ran Chen[4], Patrick Ching-Ho Hsieh[1,2*] and Klim King[3*]

[1]Graduate Institute of Life Sciences, National Defense Medical Center, Taipei, Taiwan 114;

[2]Institute of Biomedical Sciences, Academia Sinica, Taipei, Taiwan 115;

[3]Genomics Research Center, Academia Sinica, Taipei, Taiwan 115

[4]Agricultural Biotechnology Research Center, Academia Sinica, Taipei, Taiwan 115;

To whom correspondence should be addressed:

Klim King, Ph.D.

Genomics Research Center, Academia Sinica, Taipei, Taiwan 115,

Phone: 886-921-819-527; fax: 886-2-785-8847;

Email: bmkxk@ibms.sinica.edu.tw, bmkxk@yahoo.com.

Patrick C.H. Hsieh, M.D., Ph.D.

Institute of Biomedical Sciences, Academia Sinica, Taipei, Taiwan

Institute of Medical Genomics and Proteomics, and Cardiovascular Surgery Division, National Taiwan University and Hospital, Taipei, Taiwan

Phone: 886-2-27899074; fax: 886-2-27858594

E-mail: phsieh@ibms.sinica.edu.tw

Supplementary Method

Liquid Chromatography Hybrid Mass Spectrometry (LC-MS). For plasma N55 levels measurement, blood samples were collected from the tail vein before N55 administration and after i.p. 1.8 εmol/kg of N55 5, 30 and 75 min, and were purified by ethanol precipitation for LC/MS analysis. A linear ion trap-orbitrap mass spectrometer (Orbitrap Elite, Thermo Fisher Scientific, Bremen, Germany) coupled online with a UHPLC system (ACQUITY UPLC, Waters, Milford, MA) was used to quantify plasma N55 levels. For LC-MS analysis, solvent A with 0.1% FA/2% acetonitrile (CAN) in aqueous phase and solvent B with 0.1 % FA/100% ACN were used as the mobile phase for LC separation. N55 was separated online with a reverse phase column (ACQUITY UPLC BEH C18 column, 1.7 εm, 2.1 mm × 100 mm, Waters, Milford, MA) at the flow rate of 400 εl/min using gradients of 40% solvent B at 0-1 min, 40-99% solvent B at 1-6 min. The total chromatography separation time for each of the analysis was 6 min. The mass spectrometer was operated in the positive ion mode and set to one full FT-MS scan (m/z 50-600, resolution = 15,000) and eight FT-MS product ion scans (in 15,000 resolution) for precursors of N55 (m/z =392.3165) and sulfadimethoxine (m/z = 311.0814) as internal control. The fragmentation reactions of m/z 392.3165 to 263.2378 for N55 and m/z 311.0814 to 156.0778 for sulfadimethoxine were selected for quantitation. The absolute abundances of N55 were analyzed by Xcalibur software (Thermo, USA) and calculated based on the standard curve.

DPP4 activity measurement. DPP4 activity were determined by the DPP4 Inhibitor

Screening Kit (MAK203, Sigma-Aldrich, St. Louis, MO, USA) using 0.5 εL of DPP4 enzyme solution for each reaction according to the manufacturerôs protocols and then were analysed using a dual monochromator microplate fluorometer (Spectra Max Gemini EM, Molecular Devices, USA).

Supplementary Figure

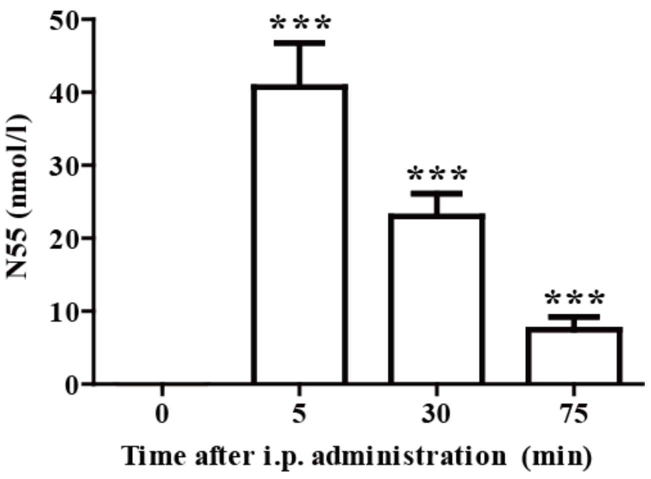

Supplementary Fig. S1. Plasma levels of N55 5, 30 and 75 min after i.p. administration of 1.8 μmol/kg of N55. Blood samples were collected from the tail vein before and after i.p. 1.8 εmol/kg of N55 5, 30 and 75 min, and then were purified by ethanol precipitation and measured by LC/MS. Values are mean ± SEM for groups of five mice. ****P* < 0.001 were comparisons of indicated time after i.p. administration vs control.

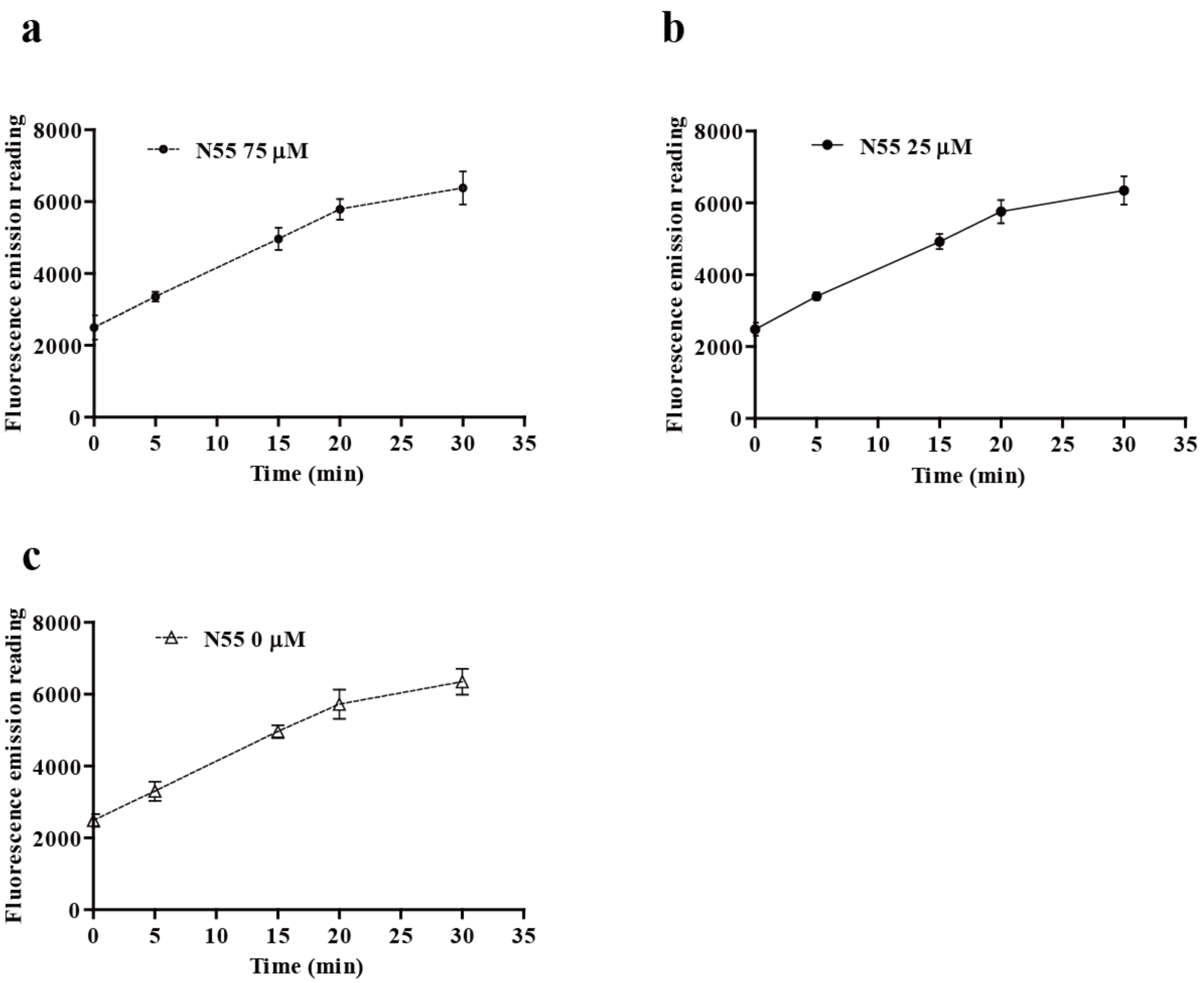

Supplementary Fig. S2. N55 did not affect the activity of DPP4. Effect of (**a**) 75 εM, (**b**) 25 εM and (**c**) 0 εM of N55 on the activity of DPP4. Values are means ± SEM of duplicate assays from three independent experiments.
